# Standardising policy and technology responses in the immediate aftermath of a pandemic: a comparative and conceptual framework

**DOI:** 10.1186/s12961-022-00951-x

**Published:** 2023-01-25

**Authors:** Naomi Moy, Marcello Antonini, Mattias Kyhlstedt, Gianluca Fiorentini, Francesco Paolucci

**Affiliations:** 1 Department of Sociology and Business Law, University of Bologna, Strada Maggiore 45, 40126 Bologna, Italy; 2 School of Medicine and Public Health, University of Newcastle, University Dr , Callaghan, NSW 2308 Australia; 3 Synergus RWE, Stationsvägen 18, 184 50 Åkersberga, Sweden; 4 Department of Economics, University of Bologna, Piazza Scaravilli 2, 40126 Bologna, Italy; 5 Newcastle Business School, University of Newcastle, Hunter St &, Auckland St, Newcastle, NSW 2300 Australia; 6 Present Address: Centre for Behavioural Economics, Society and Technology, Queensland University of Technology, 2 George Street, Brisbane, QLD 4000 Australia

**Keywords:** COVID-19, Health research systems, Policy categorisation, Public health crisis, Policy gradient, Policy interventions, Health outcomes, Economic outcomes

## Abstract

**Background:**

The initial policy response to the COVID-19 pandemic has differed widely across countries. Such variability in government interventions has made it difficult for policymakers and health research systems to compare what has happened and the effectiveness of interventions across nations. Timely information and analysis are crucial to addressing the lag between the pandemic and government responses to implement targeted interventions to alleviate the impact of the pandemic.

**Methods:**

To examine the effect government interventions and technological responses have on epidemiological and economic outcomes, this policy paper proposes a conceptual framework that provides a qualitative taxonomy of government policy directives implemented in the immediate aftermath of a pandemic announcement and before vaccines are implementable. This framework assigns a gradient indicating the intensity and extent of the policy measures and applies the gradient to four countries that share similar institutional features but different COVID-19 experiences: Italy, New Zealand, the United Kingdom and the United States of America.

**Results:**

Using the categorisation framework allows qualitative information to be presented, and more specifically the gradient can show the dynamic impact of policy interventions on specific outcomes. We have observed that the policy categorisation described here can be used by decision-makers to examine the impacts of major viral outbreaks such as SARS-CoV-2 on health and economic outcomes over time. The framework allows for a visualisation of the frequency and comparison of dominant policies and provides a conceptual tool to assess how dominant interventions (and innovations) affect different sets of health and non-health related outcomes during the response phase to the pandemic.

**Conclusions:**

Policymakers and health researchers should converge toward an optimal set of policy interventions to minimize the costs of the pandemic (i.e., health and economic), and facilitate coordination across governance levels before effective vaccines are produced. The proposed framework provides a useful tool to direct health research system resources and build a policy benchmark for future viral outbreaks where vaccines are not readily available.

## Introduction

The COVID-19 pandemic is not the first outbreak the modern world has seen (influenza A or H1N1 was declared a pandemic in 2009) [1] nor is it the only viral disease that many nations are contending with (e.g. Zika virus, Ebola virus or Measles virus). Experience with such viral outbreaks and previously declared pandemics have shaped how governments and national health research systems respond to such health care crises [2]. Previously, government responses to viral outbreaks have focused on the repurposing of drugs, vaccine development, increased use of personal protective equipment and the implementation of behavioural change on a national level [3]. However, it is noted that the effects of these measures are conditional upon the development of effective vaccines, which is the dominant strategy for maintaining positive health and economic outcomes [4]. The SARS-CoV-2 virus necessitated significant government responses due to its high fatality rate compared to other viruses, the size of the reproductive number, the number of asymptomatic spreaders, and the previous absence of a vaccine [5, 6]. Consequently, there has been large variation in the number of policies and type of policies initiated by governments in response to the SARS-CoV-2 pandemic. This large heterogeneity has made it difficult to produce valid research outputs that inform health policy and response strategies. The level of response and what policies to implement have caused debate within and across countries. In fact, it has since been observed that how politicians interpret the negative economic impact of a pandemic significantly influences their policy response—increased role of the government (e.g. overly strict, with serious economic consequences) [7] vs. reducing the role of government without transferring the task [8]. In turn, this created significant coordination problems across different governance levels (i.e., state, regional and local levels) [9–11].

The classification of the virus from a notifiable disease to a pandemic meant governments were required to step away from the status quo and implement unusual policies. Reducing or stopping the spread of a virus in the absence of a vaccine requires rapid behavioural change [12], and a way to induce a rapid change in a population is through the swift enactment of government policies. However, the policies or recommendations of choice for a government are ruled by the overall objectives, which could be (i) mitigating or suppressing the spread of the pandemics, (ii) minimising the mortality and morbidity rates or (iii) mitigating the impact on the economy [13]. Some policy initiatives work for one government objective but not others, and potentially conflicting interventions need to be implemented to pursue multiple objectives at once [13, 14]. Similarly, some of the policy priorities are context-specific and may vary by regional needs [15]. For decision-makers and health system researchers, understanding the impact of government policy and technology interventions for SARS-CoV-2 on epidemiological and economic outcomes is difficult as they have been implemented simultaneously inside each country and with large externalities due to similar measures taken by other governments at different times. An additional level of complexity in evaluating government responses comes from the positive probability that vaccines may be developed in relatively short time, as has occurred in the SARS-CoV-2 experience. As such, some responses that seem effective in the short run (e.g., eradication), might not be as effective in targeting low levels of cases in the long run.

Here we propose a conceptual framework to classify policies across a number of categories to demonstrate the level of coherence between the policy objectives pursued by governments, both explicit (i.e. publicly announced) and implicit (i.e. deducible from the policies implemented), and the instruments/policies adopted to pursue them. When designing a policy categorisation framework, researchers can adopt different approaches as the COVID-19 experience has demonstrated. One option would be to develop a monodisciplinary framework that uses a determined rationale behind the categorisation and taxonomy of policies. Examples range from frameworks that adopt a pure public health rationale [16, 17] to those that focus on the economic/financial [18–20], governance [21, 22], scientific-technological [23], and sociological such as gender [24], education [25] dimensions of the response strategy. Alternatively, one can adopt a more holistic approach where researchers try to classify all the underlying dimensions of the policy responses, such as the COVID-19 Health Systems Response Monitor (HSRM) [26] or the Oxford COVID-19 Government Response Tracker (OxGRT) do [27]. See the Oxford Supertracker for a complete overview of policy trackers [28]. A common issue of these trackers is that most of them (with OxGRT as a notable exception) do not link health or economic (-related) policies with health or economic indicators, rather they list and describe policies. Additionally, despite these categorisations providing important information to policymakers, it has been noted that there are limited theoretical or conceptual foundations for these taxonomies [29]. We follow a different strategy and propose a multidisciplinary, generalisable, framework that categorises the policy responses using a public health-economic rationale. The framework aims to provide a simple tool to categorise policies such that policymakers, health economists and health system researchers can investigate how differences in the type and timing of the direct policy responses to the pandemic across countries affect health and economic outcomes. The framework also allows for cross-country comparison to make meaningful comparisons and trace feasible policy learnings among countries that share similar institutional settings (i.e., political and economic systems, fiscal, technological and healthcare capacity). Such a qualitative taxonomy of government policy directives allows for a better understanding and evaluation of the effectiveness of the policies implemented and to identify national research priorities in the immediate periods after a pandemic is declared and before immunization is possible. In addition, we make use of a gradient that captures the intensity and extent of the policy initiatives within the categorisation framework and in relation to different health and economic objectives. Using such a system allows one to identify the dominant—more intense—pandemic directed policies during a given period and link them to specific outcomes across countries with comparable institutional settings. Timely information and analysis are crucial in addressing the gaps between a pandemic outbreak and the implementation of government interventions to alleviate the health and economic impact of the pandemic.

In the following sections, we provide a background that identifies policies used in existing and previous pandemic responses. These policies targeted health and economic objectives to directly reduce the loss of lives and the negative economic consequences of a pandemic. In the paper we attempt to consistently use SARS-CoV-2 to refer to the virus and COVID-19 to refer to the disease. However, when discussing policies that target both the virus and the disease, we use SARS-CoV-2. We then establish and define the categories for the policy categorisation framework and define each categorisation. Finally, we provide examples of the categorisation process using multiple countries that share comparable institutional settings and provide a comprehensive range of policies responses, and demonstrate how our framework differs from existing policy trackers.

### Mitigating or suppressing the spread of a virus

Measures that aim to contain and mitigate the spread of a virus tend to follow the strategy of flattening the pandemic curve (continually reducing the rate of infection) or delaying the curve (shifting the peak to the right), both of which focus on the goal of reducing the risk of overwhelming the healthcare system [30]. Mitigation measures typically include non-pharmaceutical interventions that delay the arrival of a virus into a country or reduce the spread within the country. Such measures include border closure, air-travel controls, hygiene messages, social distancing measures, school closures and restricting mass gatherings [31–35]. All of which have been estimated to provide some level of mitigation of viral spread within certain parameters, although the economic costs of such interventions can be large [34].

Much of the world’s pandemic response plans were developed to contain the spread of influenza. These plans, whilst similar in their goals, vary across countries making it difficult to comparatively examine the impact of the interventions on epidemiological outcomes. The SARS-CoV-2 outbreak has been a more severe public health pandemic than more recent outbreaks such as H1N1, and as such the resulting interventions tended to have more significant macroeconomic repercussions that required fiscal interventions.

### Minimising the mortality and morbidity rates

In addition to mitigating the spread of the virus, there is an increase in the healthcare measures pursued by a country. The initial treatment of patients can be severely impacted when health systems do not have access to the appropriate resources such as personal protective equipment, or enough medical staff. As the number of cases increases, the ability of the country’s healthcare system to respond to surge capacity is tested. In addition to being potentially overwhelmed, healthcare workers are expected to work long hours and are at risk of contracting the virus, which further constrains resources [36]. In many countries, the healthcare system was predicted to be overwhelmed by patients with COVID-19 [37]. As such, many countries and non-government organisations mobilised to increase their capacity and healthcare resources to meet predicted demand. For example, the United Kingdom increased the intensive care unit (ICU) capacity by using temporary hospitals known as nightingale hospitals [38]. This was facilitated by an increase in the number of ventilator units available, which is something several countries were acquiring in response to COVID-19. Moreover, advances in technology compared to the 2009 H1N1 pandemic have meant improvements in the ability to test, trace and treat infected cases. The SARS-CoV-2 pandemic saw the development of speedier and more accurate tests, accessible GPS and non-GPS based tracing applications and the rapid study of repurposing medications to treat the virus. Such advances can have a significant impact on epidemiology and economic outcomes when an outbreak or pandemic occurs.

#### Testing

The development of health technology has made it possible to detect the presence of a viral or bacterial infection. Continual developments have meant that viral detection tests are now more reliable and accurate, which are critical for the surveillance and management of viral outbreaks. In fact, policy directives which are suggested to have the greatest influence on epidemiological outcomes are those that govern the testing criteria for a country. Previous research has observed that surveillance systems tend to be localised and passive systems, where they detected only those who are seeking medical attention [39]. If testing is restricted then the true level of contagion in the country is unknown, as would be the ability to evaluate excess mortality and recovery rates. On the other hand, unrestricted testing is only viable if it is cost-effective and practical [40]. The testing requirements can impact the timing and choice of interventions put in place to mitigate the spread of the virus. Based on modelling of SARS-CoV-2 infections in China, it was estimated that 86% of cases went undocumented between the 10-23^rd^ of January 2020, the period before travel restrictions were implemented [41].

In addition to the value added by testing in understanding the early spread of an outbreak, the recent SARS-CoV-2 pandemic has demonstrated the value of improving test designs and capabilities to determine the extent of infection in a population. Antibody or antigen tests, for instance, were developed to help determine the rate of infection in populations and developed to test sewerage systems to assist in predicting potential COVID-19 hotspots [42]. Additionally, several countries developed rapid diagnostics for SARS-CoV-2 [43]. Part of this development was a result of the urgency to diagnose cases and improve the reliability of existing tests (reduce false positives).

#### Tracing

Contact tracing is used to link contacts of confirmed cases and identify potentially infected individuals. It has been found to be effective in the initial stages of a viral outbreak when numbers are low or in the later stages of an outbreak when spikes are occurring [44]. Continual use of effective contract tracing allows for ongoing monitoring and control of local outbreaks, which enabled the adjustment of other interventions. However, Eames and Keeling observed that when outbreaks are airborne in nature, contact tracing needs to be far more efficient and more rapid than what occurred in the past [44]. If the rate of infection is greater than the ability to trace it, containing the spread would require additional containment measures [44].

In response to the rapid spread of the SARS-CoV-2 virus, a number of countries initiated technological contact tracing methods that would speed up the process of locating potentially infected persons. One notable technological tracing intervention occurred in South Korea with the adjustment to legislation for the collection of location data, immigration records, closed circuit television recordings, banking and public transport transactions, health and medical records, and government identifying information [45]. Another was the decentralised tracking application TraceTogether in Singapore [46].

#### Treating

Initial measures to respond to treating infected persons are the use of personal protective equipment and single-patient dedicated medical equipment, isolation (if available, airborne infection isolation rooms should be used for patients who will be undergoing any aerosol-generating procedures) and strict monitoring [47]. In addition, advancing medical technologies have provided opportunities to increase the standard of medical care given to infected persons. In response to the SARS-CoV-2 pandemic, scholars and practitioners pursued advancements in care such as adjusted ventilator designs or the repurposing of drugs for the treatment of COVID-19 symptoms [48, 49]. However, the ultimate health advancement and most dominant policy intervention is the development of effective vaccine(s). Considering a nation’s target for positive health and economic outcomes, a vaccine represents the gold standard for treating viral infections. It not only provides the population with immunization, ensuring reduced mortality and pressure on the hospital systems, but it allows governments to ease restrictions thus boosting the national economy [4].

### Mitigating the economic impact

A viral outbreak or pandemic response can have a severe impact on an economy or a specific sector, such as industries with particular links to the spread of epidemics like tourism or agriculture [50, 51]. The level of a pandemic or viral outbreak and the responses not only influence demand side behaviour but can impact supply in terms of labour and products. A study in Australia in 2010 of influenza-like illnesses in childcare centres observed an increased economic burden on staff and parents, with staff losing an average of 13 h of work while having two working parents increased the economic burden due to time being needed to take off to care for a sick child [52]. In a different study estimating the economic costs of an influenza pandemic in the USA, it was estimated a greater loss occurred when a vaccine was unavailable (USD$45.3 bn) compared to when there was one (loss of USD$34.4 bn) [53].

Significant economic stimulus packages were deployed in response to the economic impacts of the COVID-19 pandemic, ranging from support for individual industries (e.g. aviation), to whole country packages designed to reduce the impact of government-initiated pandemic regulations on the economy. One example is New Zealand, which is estimated to have lost NZD$10 billion as a result of lockdowns (where all but essential industries were closed) used in response to the SARS-CoV-2 virus [54]. However, in an effort to mitigate this impact the New Zealand government introduced a NZD$12 billion economic stimulus package that targeted industries and employment [55]. Notably, the European Union for the first time suspended the fiscal compact and launched the Pandemic Emergency Purchase Programme (PEPP) which will have an overall envelope of €750 billion [56].

## Materials and methods

Our framework *"Categorising Policy & Technology Interventions (CPTI) to a viral outbreak"* provides a conceptual and comparative structure that facilitates the investigation of policies, which are implemented in the period after the pandemic declaration and before effective vaccines are available, and their impact on pandemic health and non-health related outcomes. The objective is to systematically compare the effect of policy interventions within a specific category across countries that share institutionally comparable settings (namely the political and economic systems, the fiscal and technological capacity, healthcare system development, etc.,). This enabled the investigation of the rationality and proportionality of the interventions used compared to the policy objectives, as well as the identification of research priorities at the country level.

Based on existing literature and identifiable trends in government responses to the SARS-CoV-2 pandemic worldwide, we used the following categorisations for interventions:

1. *Policy interventions to contain the spread of the virus*: these interventions focus on containment, mitigation and elimination practices to change behaviours.
2. *Policy interventions for prevention and care*: these interventions focus on the country’s healthcare system and in particular on the resourcing ability to treat active cases.
3. *Policy interventions to reduce the economic impact of containment measures*: these are fiscal interventions used to reduce the economic impact of the pandemic.

As health technology advancements have played a central role in the response strategy of many countries and health technology is continuously evolving [2], we created a health technology category:

4. *Health technology interventions*: these are the innovative technological response of industry and governments and health research systems to assist in testing, tracing and treating individuals with the virus. All focus on health monitoring and potential pharmaceutical treatments for viruses.

Compared to the existing indices and categorisation tracking systems, we use a categorisation system that implements an underlying gradient to account for the significance and the invasiveness of an intervention. The novelty of the proposed framework is that it focuses on the dominant COVID-19 intervention for containment, prevention and care and economic measures and a continuum of the health technology interventions for each period rather than on some combination of all the main policy interventions implemented. This confers a robust conceptual foundation for our taxonomy and enables the investigation of how differences in the type and timing of the direct policy responses to the pandemic across countries affect health and economic outcomes. Indeed, the specific interventions upon which the dominance criteria and their incremental levels are based reflect the outcome of the systematic mapping of policy interventions that were conducted by considering global experiences. Therefore, it is the generalisable and flexible nature of the dominance criteria that is the key contribution of our framework. While the categorisation proposed can be applied to most of the countries worldwide, cross-countries comparisons and analysis using the CPTI should be limited to countries with similar institutional settings (e.g., high-income countries, relatively free-market systems, etc.) in order to limit biases that may alter the relationship between the policy responses and the outcomes of interests [2, 57]. The reader can think, for example, to the different technological capacity available across countries. High-income countries have a larger range of technological means that can rapidly be implemented compared to middle-low-income countries. Same applies to economic and preventive means, such as hospital and treating capacity. By comparing countries with similar institutional settings, the CPTI allows for meaningful cross-countries comparisons that can set feasible policy learnings that can be rapidly implemented. Additionally, this overcomes the issue of policy trackers and indexes that erase differences in a state’s capacity to enforce measures over its entire territory or increase public spending and healthcare capacity when it comes to make cross-countries comparisons [29]. This not only allows for a more specific evaluation of the effectiveness of each intervention, but it provides a tool for policymakers to establish research priorities in areas where the country’s pandemic response strategy is weak, and a tool for researchers to observe health system priorities across countries over time.

Excluding the categories for health technology, government measures are separated into periods of escalation and de-escalation in response to case numbers. This enables the identification of the moment in which governments escalate or de-escalate their interventions for a specific group of policies and objectives. To establish the gradients and continuum for each of the categories, the potential interventions were grouped based on a similar level of characteristics.

**Fig. 1:**
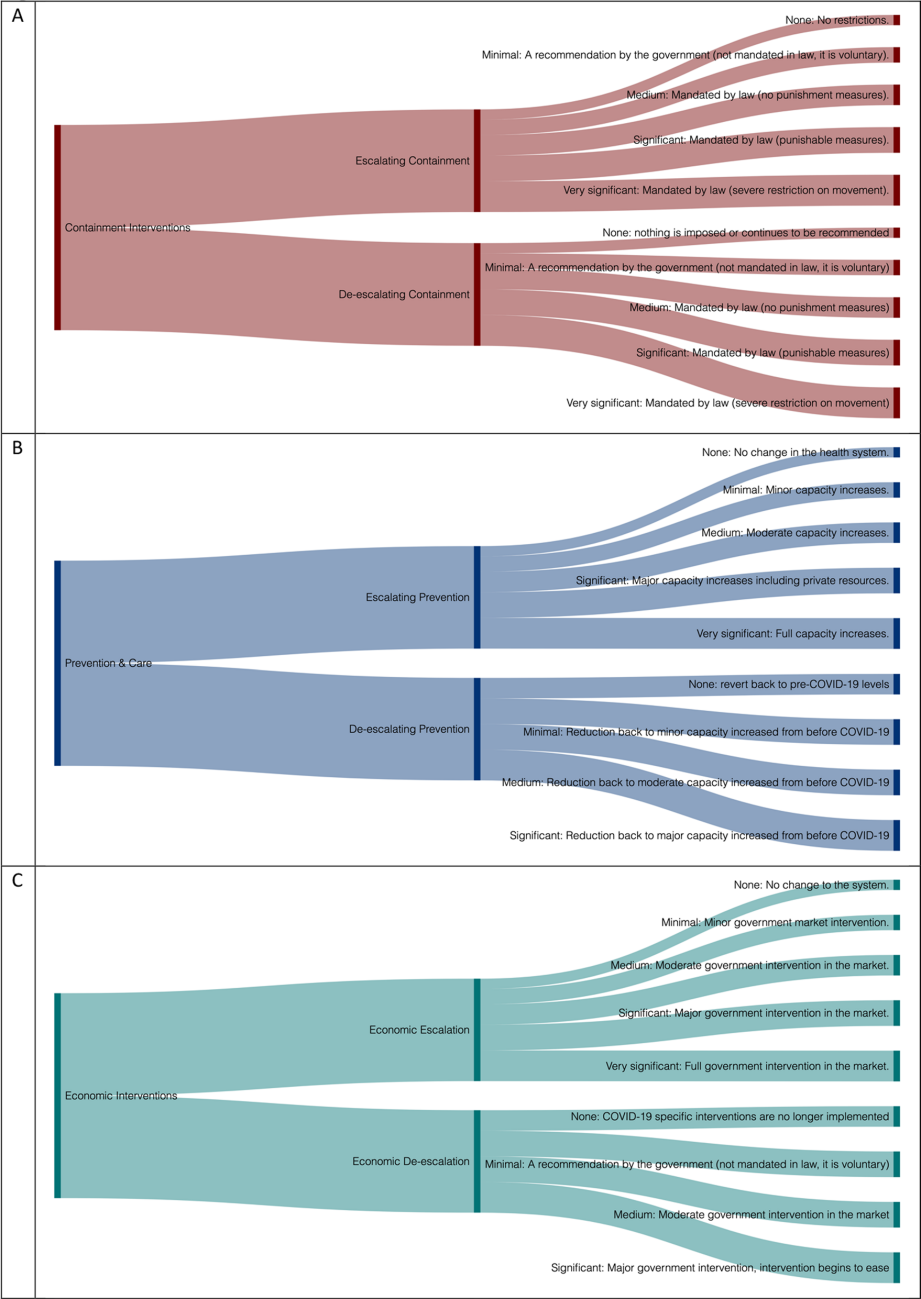

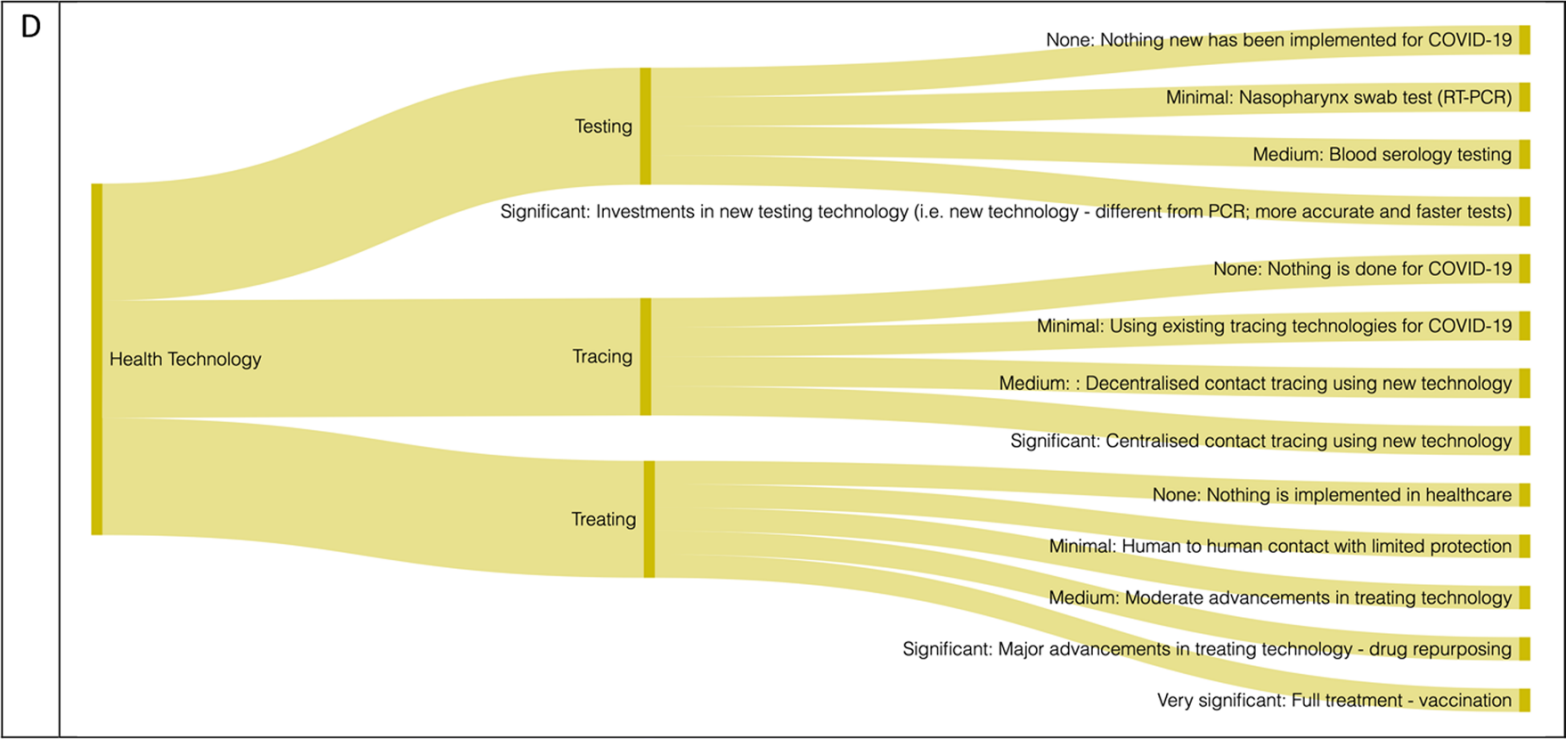
CPTI Framework. Panel **A** is for containment interventions, Panel **B** demonstrates prevention & care, Panel **C** is for economic interventions, and **D** indicates the Health Technology of the CPTI framework. See Appendix for detailed examples on the gradient and continuum

The gradient is classified according to a criterion of dominance that rules the intervention category. For instance, in the category that refers to policies aimed at mitigating the spread of the disease the gradient is based on the level of limitations to individual freedoms, in the category for policies aimed at reducing the mortality and morbidity risks, the gradient is based on the level of healthcare capacity dedicated to fight the pandemic. In the category of the policies that focus on mitigating the impact on the economy, the gradient is based on the level of government financial and regulatory interventions. For the health technology category, the sub-categories are based on a continuum of technology advancement. The initial classification is *no intervention* which is the maintenance of the initial status quo (e.g. standard medical procedure, no restrictions). The other levels of the gradient or continuum are established by identifying a dominant intervention that works as a domineer for less significant or invasive interventions in the same category or the first-stage technology used in the health technology response and in the case of tracing, this depended on the invasiveness of the technology. These gradient/continuum levels are classified as none (0); minimum (1); medium (2); significant (3), very significant (4). The full categorisation is demonstrated in Fig. 1 and Appendix.

For the *policy intervention to contain the spread of the virus*, the specifications are based on all policies that aim to contain and mitigate the spread of COVID-19. The gradient for this categorisation is based on the level of limitations to individual mobility. A *minimal* intervention for containment interventions is one which is not mandated by law, such as a government recommendation to work at home if possible, and hygiene messages. *Medium* interventions for containment are those which are mandated by law, however, no fines are imposed to enforce behaviour. For example, the closing of schools or the declaration of a state of emergency. *Significant* measures for containment include those that are mandated by law and enforced, such as the closure of borders, restrictions on social distancing or enforced quarantines. Finally, *very significant* interventions for containing the spread are the complete restrictions on movement on the populace and all non-essential industries are shutdown. More examples are provided in Appendix.

The gradients for the *prevention and care interventions* are based on the degree of public healthcare system takeover. This category is based on the evidence that all healthcare systems are hybrid considering the financing and provision of the services. In response to the pandemic, even countries where a tax-funded National Healthcare Systems model prevails (e.g. UK and Italy), secured private facilities to face the peak of contagions and hospitalisations [58–60]. The gradient ranges from no changes to the status quo (*none*, being no change) of the existing healthcare system to all healthcare related resources (*very significant*) devoted to the public healthcare system to respond to COVID-19. *Minimal* health care interventions include those which increase the capacity of the healthcare system such as provision of additional healthcare equipment, while *medium* capacity increases are those that affect medical staff, such as redeployment or early graduation of eligible students. A *significant* response is one that has a larger impact on the healthcare system and incorporates private healthcare resources, such as the suspension of elective services or the use of private facilities for provision of public healthcare. In conclusion, a *very significant* response entails the public healthcare system takeover, in which all resources (e.g., staff, facilities, beds and equipment) are devoted to a direct response to the outbreak and significant prevention and care interventions such as the suspension of elective surgery and the suspension of private insurance premiums are enacted.

The dominance criterion for the *economic impact* is based on the degree of government intervention in the economy and the regulator’s interference in the market. It ranges from no changes to the status quo (no economic interventions instigated by COVID-19) to the suspension of the free market and shift towards a centrally planned economy. This level of intervention has not been observed in any nation, but would be the upper limit for economic interventions. A minimal policy is one that has a minor market intervention, where smaller and more individualised funds are provided to individuals. This escalates into medium and significant gradients that dominate this category, where the level of government intervention into the market has increased and where the government subsidies become more widespread (i.e. extraordinary increase of public spending for industry bailout and quantitative easing).

The continuum for the *health technology* is determined by the advancement of technology. For example, the health technology testing gradient starts off with none, then progresses to the existing standardised nasopharynx swab test based on the real-time reverse transcriptase-polymerase chain reaction (RT-PCR) technology test for COVID-19 which confirms cases, followed by the use of serology to determine the share of the population affected. Finally, there is increased investment to expand existing technologies used to test and determine in the shortest possible time the presence of SARS-CoV-2, which is the domineering intervention for this category [50]. A similar gradient is followed for the tracings and treating technologies.

It is worth underlining here that there are significant operational challenges in developing policy gradients that can fit all countries' experiences due to the heterogeneity of their institutional settings. Therefore, we made an operational choice in the definition of the dominance criteria that allows the framework to apply to most countries worldwide. Despite being the result of a systematic mapping of interventions worldwide, we do acknowledge, however, that the framework might not capture homogenously the full spectrum of countries' experiences. An example that clarifies this to the reader is the dominance criterion for economic interventions, which is based on the degree of government intervention in the economy and the regulator’s interference in the market. Whilst the vast majority of the countries in the world allocate resources by means of market systems in many, if not most areas, there is a restricted group of countries that present a centrally planned economic system. For these restricted group of countries, adjustments to the dominance criterion should be implemented to apply the framework consistently. Such clarification is also useful to remark once more the importance of applying the CPTI between countries with similar institutional settings (e.g., high-income countries, relatively free-market systems, high hospital and technological capacity, etc.) in order to limit biases that may alter the relationship between the policy responses and the outcomes of interests and draw timely and feasible policy learnings.

### Generalisability and application for future pandemic outbreaks

The strength of the CPTI framework relies on the nature of the dominance criteria that enables both a robust conceptual foundation and a simple categorisation mechanism. This allows the framework to be generally applicable across countries, and viral outbreaks with characteristics that differ from those of COVID-19. Although the CPTI framework was developed using airborne transmission viruses (i.e. SARS-CoV-2) as the reference for the health policy and technology interventions, policymakers and health system researchers can adapt the framework by classifying the interventions that best fit the potential response to cope with the virus at hand. Indeed, the dominance criteria proposed here (synthetically reported in the Appendix) are not anchored to the virus itself but rather to the intrinsic nature of containment interventions (preventive, economic and technological) and their incremental intensity. This makes the framework a benchmark that can be adjusted and adapted to different pandemic contexts. For example, if we consider the health technology gradient, the examples reported in the various levels might not completely fit the required intervention for waterborne diseases, soil-transmitted diseases or bloodborne diseases. For the first two categories PCR and rapid tests can be used as an alternative to the confirmation by cell culture (i.e., cholera diagnosis [61]). For the latter virus typologies, nasal swabs or PCR tests do not apply. Nevertheless, the dominance criterion suggested, together with the categorisation of each level, facilitates the classification of the policy interventions. Starting from the status quo, that is the current set of tools to treat, test or trace a disease, researchers can classify the incremental interventions once they are enacted, following the logic set by the dominance criteria.

## Results

**Fig. 2:**
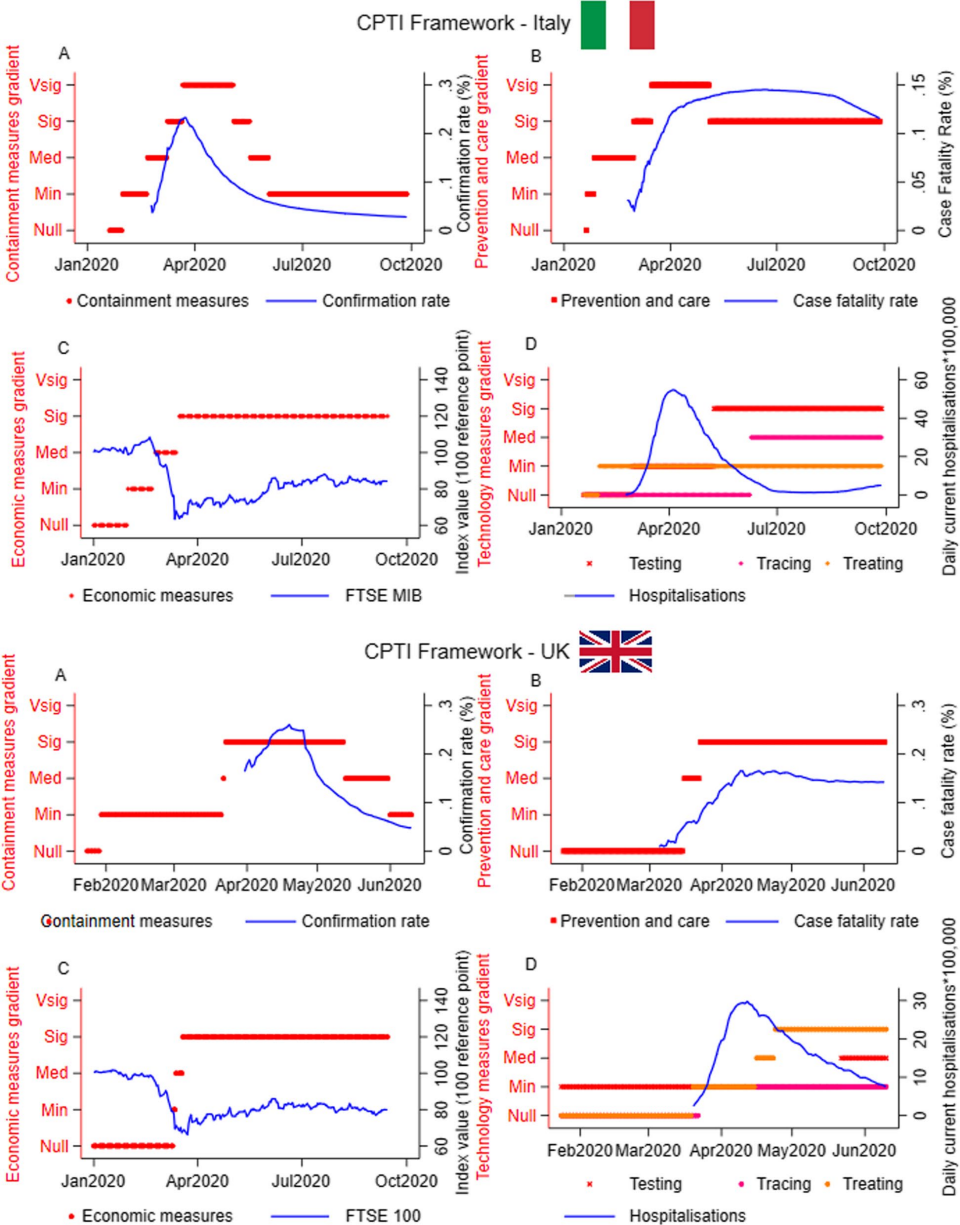
Application of the CPTI framework in Italy and the UK. Panel **A**: containment measures gradient, Panel **B**: prevention and care gradient; Panel **C**: economic measures gradient; Panel **D**: health technology measures gradient. The graphs are not to be intended as a tool to identify correlation between the level of the policies and the outcome of interests. The levels of the gradients in the y-axis are ordinal measures. Min: Minimal; Med: Medium; Sig: Significant; Vsig: Very significant

**Fig. 3:**
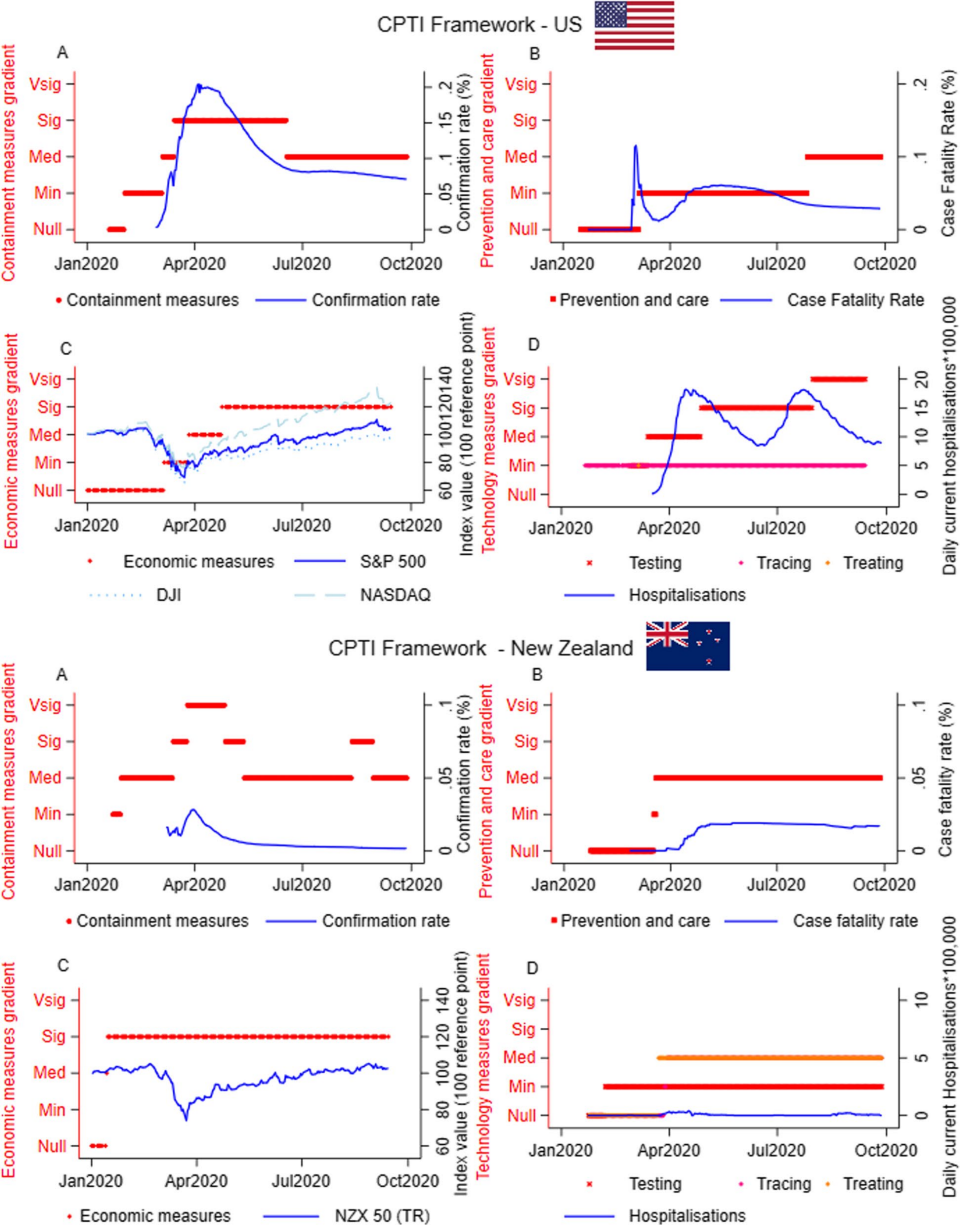
Application of the CPTI framework in the US and New Zealand. Panel **A**: containment measures gradient, Panel **B**: prevention and care gradient; Panel **C**: economic measures gradient; Panel **D**: health technology measures gradient. The treating curve in the technology gradient is not reported for US due to the large variability among States. The graphs are not to be intended as a tool to identify correlation between the level of the policies and the outcome of interests. The levels of the gradients in the y-axis are ordinal measures. Min: Minimal; Med: Medium; Sig: Significant; Vsig: Very significant

The categorisation framework can be used to present information qualitatively as reported in Benitez et al. [62]. Additionally, the gradient can be used to show the dynamic impact of policy interventions on specific outcomes ranging from the most common economic and health outcomes (i.e., fatality rate, ICU saturation, stock market index, etc.; Figs. 2, 3) to more political and/or social outcomes (i.e., interpersonal violence and abuse, substance abuse, educational outcomes, changes in general trust for the government or the public health authorities, etc.,). See Berardi et al. [58] and Fouda et al. [63] for some examples. Indeed, the flexibility of the CPTI framework and its gradient allows researchers to focus on the outcome of interest. One note, however, is that there are practical limitations in focusing on these additional outcomes as the availability of comparable and reliable data varies across countries. In some cases, information was typically disclosed with a time lag (i.e., quarterly or even annually). Therefore, scholars and policymakers must evaluate the scope of their analysis and choose the most suitable data to focus on. It is important also to underline that the graphical visualisation of the framework with the outcome of interest does not (and cannot) serve as a tool to identify correlations between the policy enacted and the outcome of interests. Rather, it is a flexible and useful tool for policymakers and health system researchers to identify the policies that may have a positive impact on the underlying outcomes of interest and for which further, and more statistically accurate, examinations must be conducted to establish the degree of correlation with the outcomes.

In this paper, we prioritise health and economic outcome over other possible outcomes because these data are more readily available in the immediate aftermath and have played a critical role in political and scientific debates. Our aim is to provide a timely and simple tool to categorise policies such that policymakers, health economists and health system researchers can investigate how differences in the type and timing of the direct policy responses to the pandemic across countries affect health and economic outcomes.

We use data from Italy, New Zealand, the United Kingdom (UK) and the United States of America (US) to demonstrate the application of CPTI. This specific set of countries was chosen for several reasons. Firstly, they share common institutional features (i.e., democratic, market-oriented, high-income countries with a relatively large fiscal, technological and health care capacity). This allowed us to isolate our variable of interest—the type of policy response and its timing across countries—on health and non-health outcomes. Such a comparison based on institutional homogeneity (i.e., political and economic systems, fiscal, technological and healthcare system capacities) is recommended for the application of the CPTI to reduce the impact of other confounding factors that may alter the relationships between the policy responses and the outcomes of interest. Secondly, the countries chosen provide interesting variations in the mix and timing of policy responses before vaccines availability, making the comparative analysis between them an effective tool to isolate the distinctive impact of each policy on each outcome of interest holding other factors constant. For example, the four countries cover all the main policy response strategies observed worldwide before vaccines availability. New Zealand, favoured by its geographical location, adopted an elimination strategy by using strict measures to limit the spread of the disease [63]. Italy was forced to implement immediate and restrictive policies as it was the first Western country to be significantly hit by the virus and did not have the adequate resources to cope with the higher demand for hospital services caused by the virus [58]. The UK government, on the contrary, initially focused on recommendations for behaviour change in the general public, underestimating the impact of the virus until the end of March 2020 when a national lockdown was imposed [59]. On the other hand, the mitigation strategy in the US was a decentralised response, with relatively softer measures compared to the aforesaid countries, coupled with a very effective and centralised response in terms of economic stimulus and technological interventions [64]. Similarly, the choice of these countries allows to cover a wide array of political leaderships (and political majorities) that managed the policy response to SARS-CoV-2. We range from declared populist leaders (despite some notable differences) such as Donald Trump and Boris Johnson, to the markedly progressive government of Jacinta Ardern in New Zealand, passing by the centre-left coalition government led by Giuseppe Conte in Italy. This mix can reflect the political situation in other nations and therefore provide a benchmark for further applications of the framework. Lastly, the four countries provide an interesting mix of hybrid healthcare system types that reflect most of the healthcare systems worldwide, ranging from the managed competition in the US to the decentralised NHS in Italy.

Data for Italy, New Zealand, UK, and US were gathered from the respective government agencies [65–68]. Differences in state collections in the US, meant we used hospitalisation data made publicly available from “The COVID Tracking Project” [69]. The four categorisations and the subsequent gradients are applied and reported on different COVID-19 outcomes, such as the confirmation rate (positive cases/total tested), case fatality rate, daily trend of the most important stock market indices values, and the daily number of hospitalisations per 100,000 inhabitants. Variation in the interventions implemented between the four devolved administrations of the UK, meant UK data is reported until June 2020 [59]. Given the heterogeneity in the testing strategy and classifications, the outcomes reported are demonstrative and chosen to provide an overview of the use of the CPTI framework.

## Discussion

The CTPI framework identifies the weaknesses and strengths that have been attributed to the Italian COVID-19 response (Fig. 2). Despite additional directives implemented in January 2020 to prepare for COVID-19 cases, the limited results of early containment and preventive measures led to an increased tightening of containment actions, as seen by gradient movements in Fig. 2A. A similar shape can be detected for the prevention and care measures (Fig. 2B), with rapid escalation and no de-escalation until the curve of the case fatality rate was demonstrably flattening. In terms of health technology, there were null or minimal testing and tracing dimensions during the most critical phase, when the majority of cases were still active. The development of a contact tracing app and serology tests were implemented in Italy after the curve flattened (Fig. 2D) revealing weaknesses in the health research system. The effort to develop more advanced treatments has been attempted, primarily via repurposing of existing drugs and this is still an ongoing area of research. Finally, whilst the tracing App *Immuni* did not reach the expected target of downloads, an improvement in the overall tracing strategy seems the basis for a good response [70].

A similar pattern is observed in the UK and the US, where softer containment measures were originally implemented in response to SARS-CoV-2. Following the rapid escalation in the number of cases, both countries enforced a lockdown before the peak of the confirmation rate (Figs. 2E and 3A), without shutting down non-essential activities. Similarly, in the UK, preventive measures increased in strictness as deaths increased Fig. 2F), while in the US preventative measures were relatively low (Fig. 3B). This can be partly explained by the initial US healthcare system and research capacity, which compared to other countries required minimum intervention. Overall, all countries recorded a flattening of the case fatality curve after interventions on healthcare system capacity.

The UK government was more responsive in terms of COVID-19 testing strategy and treating measures (Fig. 2H). Indeed, the NHS developed a centralised coronavirus contact-tracing app which switched to an Apple and Google app [2]. In the US (Fig. 3D), testing and treating interventions were initiated as the confirmation rates peaked or before, while more stringent tracing measures were not introduced until after the peak and when cases were plateauing. Figure 3D shows that the strategy implemented by the US has not prevented a second peak of hospital admissions.

Among the countries considered, New Zealand enacted the strictest response strategy, targeting the policy objective of elimination. In line with this objective, responses in New Zealand occurred rapidly, adjusting to the strictest level as the confirmation rate of COVID-19 cases increased (Fig. 3E). This rapid response occurred both during the initial outbreak and the secondary smaller wave in August of 2020, as demonstrated by the second shift in the containment measure gradient—an immediate response occurred with the prevention and care measures (Fig. 3F). Notably, New Zealand was able to keep the preventive policies at a relatively low level, while Italy and the UK pursued stricter measures during the period covered.

Finally, the level of economic interventions has been considerable in all the countries. Although the introduction of economic interventions was gradual in Italy and the US, once initiated they were maintained despite a flattening of the curve (Figs. 2C and 3C). This trend is observable for the UK and New Zealand (Figs. 2G and 3G) which both enacted a quicker escalating economic strategy. However, most of the economic interventions adopted are short-term policies, and governments committed to implement them over a longer period of time to avoid an economic collapse. Accordingly, the expectations of future profits increased after the shock. This demonstrates the significant government effort to mitigate the economic downturn being faced in the short and medium term. Nevertheless, only the US and New Zealand stock markets have been able to recover to their level pre-SARS-CoV-2. For the US (see Fig. 2C), this might be attributed to the level of multinational corporations whose value depends on worldwide expectations, whilst for New Zealand the expectations for future profits might have been positively influenced by the effective response strategy enacted by the government.

While we acknowledge that the stock market indices do not fully reflect the underlying status of the economy, however, they provide a good approximation of the overall investors’ confidence. In periods characterised by exceptional uncertainty and where policymakers must make rapid and informed decisions, the availability of real-time data is critical for the appropriate information to be considered. Macroeconomic data are disclosed with a significant lag, and there is a delay in research output to provide support to policymakers. On the other hand, information about the investors’ confidence is immediately available and more sensitive to daily changes and might be regarded as a relevant indicator of the pandemic's impact and the related policy responses on consumers and investors. Indeed, a higher investors’ confidence and rising stock market indexes are expected to produce more demand for consumer goods and positive financial conditions for the companies on the markets that can issue new shares to gain capital to start new projects, make new investments and hire workers, ultimately favouring the overall economy. On the contrary, negative confidence and stock index trends are expected to reduce spending, making more challenging for the companies to collect capital in the market, reducing sales and overall revenues. The reader should note that the application proposed here might allow for other, more appropriate, economic indicators, if and when data becomes available. Finally, we note that the epidemiological curves we observe in Figs. 2, 3 could be driven by factors outside the scope of interventions, e.g. seasonality of coronaviruses.

## Conclusions

We have observed that the policy categorisation described here can be used by decision-makers and health system researchers to examine the impact of SARS-CoV-2 on health and economic outcomes over time. Not only does the framework allow for a visualisation of the frequency and comparison of dominant policies, but it provides an overview of how dominant interventions (and innovations) affect different health and non-health related outcomes during the response phase of the SARS-CoV-2 pandemic. This is critical to directing health research system resources and building a policy benchmark for future strains of SARS-CoV-2 and other viral outbreaks where vaccines are not readily available. The policy framework can also serve to inform policymakers and public health practitioners on the policies that facilitate a “controlled” transition of the virus from epidemic to endemic with or without vaccine availability. Further, we contribute to the existing literature on policy trackers and indexes by providing an objective ordinal threshold through the gradient (*None to Very significant*) which can inform policymakers who decide on response strategies or when to implement a given intervention (as the stringency indexes do), and the ability to group and describe the different nature of the interventions implemented to inform the general public (as the policy trackers do).

This is particularly relevant as ultimately the convergence to an optimal set of policy interventions before vaccine availability would involve mitigating, preventive, technological and economic interventions that are connected to the policy objectives set by governments and to the virus characteristics or level of viral spread. Such a convergence on optimal policies could provide a useful tool to assist governments in overcoming the instability in the policy process and guide the health research agenda before immunization is implementable. This, in its turn, might reduce the risk that the policy process is driven mainly by the necessity to delay negative consequences [8] rather than to pursue specific policy objectives that could have avoided the negative outcomes in the first place (i.e., dynamic lockdown in hot spots).

An additional strength of the CPTI classification is that it can be applied at every governance level (i.e., national, regional, local) and it does not require advanced quantitative skills as indexes do for example. Therefore, it can foster interdisciplinary collaborations and overcome the research fragmentation that exists between health research, policy and systems. Such an interdisciplinary approach is crucial to control the SARS-CoV-2 pandemic and strengthen the effectiveness of national health research systems to manage future epidemics and public health crises [71]. Future research can validate the framework by applying the categorisation and the gradient to countries with different institutional settings (e.g., developing countries, authoritarian countries, etc.). Additionally, future research can apply the CPTI gradient to investigate the effect of the policy mix on a range of social and political outcomes that are critical in the overall evaluation of a response strategy to the pandemic. Examples range from changes in interpersonal violence and abuse, substance abuse, educational outcomes, and mental health outcomes to changes in general trust in the government and public health authorities. In this paper we purposely focused on a limited set of health and economic outcomes to demonstrate the framework's application to data that are available and that are used by policymakers to make real-time decisions.

The framework is limited as it does not address the impact of the overall strategy used by the governments to respond to SARS-CoV-2. Instead, the policy categorisation process provides insight into the immediate response to SARS-CoV-2. For instance, New Zealand focused on an elimination strategy, which explains why the stand-down of restrictions did not occur until the number of active cases had dropped to almost zero. However, future research could develop an assessment framework for SARS-CoV-2 strategies used by governments to manage and evaluate the outcome of policies. An additional limitation is that the application of the policy gradients did not include the few countries with a fully planned economy with no private actors in the economy and healthcare systems. Future research could fill this gap by applying—with some adjustments—the gradients to these countries. In addition, the CPTI framework is not a policy tracker: instead of tracking policies, it provides a structure in which current and future trackers can group government and responses to pandemics for further analysis. As such, the CPTI provides a qualitative taxonomy of different interventions which can contribute to future modelling that uses richer and more informed data when it becomes available Whilst the proposed gradient is conceived to evaluate the initial policy response phase of a viral outbreak, it can enable the observation of the impact of vaccine marketisation on the policy interventions over time. Finally, the gradients for health technology could be expanded upon, as current and future technological developments will mean that successive waves of SARS-CoV-2 would start from the current minimal category rather than none.

## Data Availability

The datasets used and/or analysed during the current study are available from the corresponding author on reasonable request.

## Appendix

See Table 1.

**Table 1:** CPTI framework

Categorisation	Sub-category	Gradient	Definition/Example
Policy interventions to contain the spread of the virus (Behaviour, containment, mitigation)	Escalating interventions	None	Main: No restriction (maintaining the status quo)
Dominance: free movement restriction			
		Minimal (1)	A recommendation by the government (not mandated in law, it is voluntary). Main: work from home if possible
			Complementary: advised against travel to specific regions, advised to use masks, wash your hands, website launch of information about the virus, launch of phone helplines, temperature checks at the border
		Medium (2)	Mandated by law (no punishment measures). Main: Closing schools, closing of high street/retail stores *plus* minimal interventions
			Complementary: self-quarantine, state of disaster declared, self-cancelling of events, certification of fitness for travel, advised against all travel overseas. Increased testing to wider public
		Significant (3)	Mandated by law (punishable measures). Main: borders closing (national, regional) plus medium interventions
			Complementary: Enforced quarantine, enforced social distancing, gatherings, mandatory masks, restriction for bars, restaurants and self-care
		Very Significant (4)	Mandated by law (sever restriction on movement). Main: Enforce lockdown and shut down of non-essential economic activities *plus* significant interventions
	De-escalating interventions (implementing interventions that begin to reduce for phase one)	Significant (3)	Mandated by law (punishable measures). Main: borders closing (national, regional)
			Complementary: Enforced quarantine, enforced social distancing, gatherings, mandatory masks, restriction for bars, restaurants and self-care
		Medium (2)	Mandated by law (no punishment measures). Main: Re-opening schools, re-opening of high street/retail stores (social activities)
			Complementary: self-quarantine, reducing the state of disaster declared, removing requirements for certification of fitness for travel, adjusting/reducing advise against all travel overseas
		Minimal (1)	A recommendation by the government (not mandated in law, it is voluntary). Main: work from home if possible
			Complementary: restaurants and gyms reopened, social distancing measures in terms of washing hands, working from home if possible are still maintained
		None	Nothing is imposed or continues to be recommended
Policy interventions for prevention and care Dominance: Public healthcare system takeover. All resources devoted to the public healthcare system	Escalating interventions	None	Main: healthcare system experiences no change
		Minimal (1)	Minor capacity increases. Main: Increased capacity of the public system
			E.g. Additional PPE, Increased spending on PPE or beds, adjustments to border controls for medical goods arriving into the country, flu vaccine drive
		Medium (2)	Moderate capacity increases. Main: government callout for health resources from industry for a better response (ventilators, masks, retired and student medical professionals, relaxation of medical staff contracting requirements, etc.) plus minimal prevention and care interventions
			Complementary: clinical trial announced for vaccine within country
		Significant (3)	Major capacity increases including private resources Main: partial takeover of the private system plus medium prevention and care interventions
			E.g. temporary hospitals, addition of beds, supply of ventilators, new workforce
			Complementary: retired workers coming out of retirement and working
		Very significant (4)	Full capacity increases. Main: All the resources in the healthcare system are devoted to the public healthcare system plus significant prevention and care interventions
			E.g. suspension of elective surgery in private facilities, suspension of private insurance premiums
	De-escalating interventions (implementing interventions that begin to reduce for phase one)	Significant (3)	Reduction back to major capacity increased from before COVID-19. Main: partial takeover of the private system
			E.g. temporary hospitals, addition of beds, supply of ventilators, new workforce
			Complementary: retired workers coming out of retirement/students no longer required
		Medium (2)	Reduction back to moderate capacity increased from before COVID-19. Main: government callout for health resources from industry for a better response (ventilators, masks, retired and student medical professionals, relaxation of medical staff contracting requirements, etc.)
			Complementary: clinical trial announced for vaccine within country
		Minimal (1)	Reduction back to minor capacity increased from before COVID-19. Main: Increased capacity of the public system
			E.g. Additional PPE (spending/, adjustments to border controls for medical goods arriving into the country
		None (0)	Reversion back to pre-COVID-19 levels
Policy interventions to reduce the economic impact Dominance: degree of intervention of the government in the economy. Push toward central planned economy [increased intervention in the market]	Escalating Interventions: Based on the size and the extent of the stimulus package that is implemented as a result of the pandemic	None (0)	No change to the economic system
		Minimal (1)	Minor market intervention by the government. Main: Bonuses, credits, tax relief/deferrals from the state/county/council level, rent freezes
		Medium (2)	Moderate government intervention in the market. Main: Funding for specific industries or sectors and specific individuals (families or sole traders only) *plus* minimal economic interventions
			E.g. Funding targeting education, health resources, agriculture, financial sector, education (e.g. schools and higher education), technology sector, energy sector etc Complementary: allowing movement of workers to meet demand (Germany allowing foreign workers in to help pick produce)
		Significant (3)	Major government intervention in the market. Main: Extraordinary increase of public spending for industry bailout and quantitative easing *plus* medium economic interventions
			E.g. Industry bailout (large funds), relief cheques, liquidity, interest rate adjustments, wage subsidisation
		Very significant (4)	Full government intervention in the market. Main: Suspension of free-market and shift toward central planned economy
			E.g. all the industries of the country are devoted to produce or assist the emergency plan imposed by the government, exports suspended
	De-escalating interventions	Significant (3)	Major government intervention, intervention begins to ease*.* Main: Extraordinary increase of public spending for industry bailout and quantitative easing
			E.g. Industry bailout (large funds), relief cheques, liquidity, interest rate adjustments
		Medium (2)	Moderate government intervention in the market. Main: Funding for specific industries or sectors and specific tax relief (families or sole traders only) eased/reduced
			E.g. the funding targeting education, health resources, agriculture, financial sector, higher education, technology sector, energy sector etc., Complementary: allowing movement of workers to meet demand (Germany allowing foreign workers in to help pick produce)
		Minimal (1)	Minor government market intervention. Main: Bonuses, credits, tax referrals from the state
		None (0)	COVID-19 specific interventions are no longer implemented
Health Technology Announcement or use of health technology Categorisation is based on the size and invasiveness/extent of the technology	Testing Objective based criteria: confirming, exploring, expanding	None (0)	Nothing new has been implemented for COVID-19
		Minimal (1)	Nasopharynx swab test (RT-PCR)
		Medium (2)	Blood serology testing
		Significant (3)	Investments in new testing technology (i.e. new technology—different from PCR; more accurate and faster tests)
	Tracing Degree of invasiveness of the technology	None (0)	Nothing is done for COVID-19
		Minimal (1)	Main: Using existing tracing technologies for COVID-19
			E.g. Use of call centres; Symptom tracker app; COVID-19 tracker teams
		Medium (2)	Main: Decentralised contact tracing using new technology E.g. Contact tracing through GPS/Bluetooth tracking (not provided to government), detecting COVID-19 in sewage
		Significant (3)	Main: Centralised contact tracing using new technology
			E.g. Contact tracing through GPS/Bluetooth tracking (provided to government), Artificial intelligence to detect population risk groups
	Treating The extent of advancement of technology	None (0)	Human to human contact, standard ICU, no use of PPE etc. for COVID-19
		Minimal (1)	Main: Human to human contact with limited protection
			E.g. Use of PPE; temperature checks;
		Medium (2)	Main: Intensive/Hospital care: respirator advancements (new modifications and types); technology use for managing viral cases (new apps etc.); extensive telehealth
		Significant (3)	Main: Drug repurposing (proven outcomes); robots; compulsory use of telehealth
		Very Significant (4)	Main: Vaccine
